# An epidemiological study assessing the prevalence of human papillomavirus types in women in the Kingdom of Bahrain

**DOI:** 10.1186/1471-2407-14-905

**Published:** 2014-12-03

**Authors:** Khairya Moosa, Adel Salman Alsayyad, Wim Quint, Kusuma Gopala, Rodrigo DeAntonio

**Affiliations:** Arabian Gulf University/Medical College, Manama, Bahrain; Chief of Disease Control Section, Ministry of Health, Manama, Bahrain; DDL Diagnostic Laboratory, Rijswijk, the Netherlands; GlaxoSmithKline Pharmaceuticals Ltd., Bangalore, India; GlaxoSmithKline Vaccines, Wavre, Belgium

**Keywords:** Epidemiology, Human papillomavirus, Kingdom of Bahrain, Prevalence, Type distribution

## Abstract

**Background:**

Persistent infection with high-risk (HR) human papillomavirus (HPV) causes cervical cancer, the fourth most frequent cancer in the Kingdom of Bahrain, with an annual incidence of four per 100,000 women. The aim of this study was to assess the prevalence and type distribution of HPV in Bahraini and non-Bahraini women attending routine screening. HPV prevalence was assessed by risk factors and age distribution. Health-related behaviors and HPV awareness were also studied.

**Methods:**

This observational study was conducted between October 2010 and November 2011 in the Kingdom of Bahrain (NCT01205412). Women aged either ≥20 years attending out-patient health services for routine cervical screening or ≥16 years attending post-natal check-ups were enrolled. Cervical samples were collected and tested for HPV-DNA by polymerase chain reaction and typed using the SPF_10_ DEIA/LiPA25 system. All women completed two questionnaires on health-related behavior (education level, age at first marriage, number of marital partners, parity and smoking status) and HPV infection awareness.

**Results:**

HPV DNA was detected in 56 of the 571 women included in the final analysis (9.8%); 28 (4.9%), 15 (2.6%) and 13 (2.3%) women were infected with single, multiple and unidentifiable HPV types, respectively. The most prevalent HPV types among the HPV positive women were HR-HPV-52 in eight (1.4%), HR-HPV-16, -31 and -51 in six women each (1.1%); low-risk (LR)-HPV-6 in four (0.7%); and LR-HPV-70, -74 in three women each (0.5%). Co-infection with other HR-HPV types was observed in 50% HPV-16-positive women (with HPV-31, -45 and -56) and in both HPV-18-positive women (with HPV-52). None of the health-related risk factors studied were associated with any HR-HPV infection. More than half of women (68.7%) had never heard about HPV, but most women (91.3%) in our study were interested in HPV-vaccination.

**Conclusion:**

HPV prevalence in Bahraini women was 9.8%. The most frequently observed HPV types were HR-HPV-52, -16, -31 and -51 and LR-HPV-6, -70 and -74. These are useful baseline data for health authorities to determine the potential impact of preventive measures including the use of prophylactic vaccines to reduce the burden of cervical cancer.

## Background

Globally, cervical cancer (CC) is the second most frequent cancer in women, with an estimated 1.6 million women diagnosed with CC between 2004 and 2008 [1]. In the Kingdom of Bahrain, 369,821 women aged under 15 years are at a risk of CC [2]. CC ranks as the third most frequent cause of cancer in women with a crude annual incidence of four per 100,000 women in the Kingdom of Bahrain [2–4]. Twenty two new cases of CC are diagnosed every year and CC causes approximately 5 deaths annually in the Kingdom of Bahrain [2].

It is well established that persistent infection with high-risk (HR) human papillomavirus (HPV) causes CC [5, 6]. Globally, HR-HPV types -16 and -18 are responsible for almost 70% of the overall CC cases [7], but HR-HPV types -31, -33, -35, -39, -45, -51, -52, -56, -58, -59, -68, -73 and -82; and low-risk (LR) HPV-types -6, -11, -40, -42, -43, -44, -54, -61, -70, -72, -81, and CP6108 have also been associated with the disease [6]. A previous study conducted at two medical centers in the Kingdom of Bahrain observed cervical HPV infection in approximately 11% of women [8].

Two prophylactic HPV vaccines are currently licensed in many countries: bivalent *Cervarix*® (GlaxoSmithKline, Belgium) and quadrivalent *Gardasil*® (Merck and Co., Inc., Whitehouse Station, New Jersey). Both vaccines are well tolerated with good efficacy profiles in preventing HPV infection [9–16].

As baseline data on HPV epidemiology and distribution of HPV types in Bahrain are lacking, it is not possible to accurately assess the disease burden associated with CC and it is therefore difficult to measure the impact of preventive measures, such as the introduction of vaccination. This study was designed to evaluate the prevalence and type distribution of HPV in Bahraini women. The study also evaluated HPV type distribution by risk category in women of different ages, and also documented the awareness of HPV infection, vaccination and health-related behaviors through questionnaires.

## Methods

### Study design and population

This observational, cross-sectional, study was conducted in the Kingdom of Bahrain between October 2010 and November 2011 (NCT01205412) at four primary healthcare centers (Isa town Heath Center, Arad Health Center, Budaiya Health Center, Sitra Health Center) and the American Mission Hospital. Women aged either ≥20 years undergoing routine cervical screening or ≥16 years attending post-natal check-ups and willing to provide a cervical sample were enrolled. Women were excluded for: immunosuppression, abnormal cervical samples, heavy menstrual bleeding that would interfere with screening, hysterectomy, previous HPV vaccination or pregnancy.

The study protocol was reviewed and approved by the ethical committee in the Ministry of Health. The study was conducted in accordance with the Declaration of Helsinki and Good Clinical Practice. Informed consent was obtained from all eligible women before enrollment.

### Sample collection and laboratory procedures

Endocervical samples, collected by a trained practitioner/gynecologist using a cytobrush, were preserved in *Thinprep*® (Hologic, Inc) solution and stored on-site at room temperature for four weeks before shipment at -20°C to the DDL Diagnostic Laboratory (Rijswijk, the Netherlands).

HPV-DNA isolated from cervical samples (500 μl) using the MagNA Pure LC Total NAILV kit (Roche Diagnostics, Almere, The Netherlands) and eluted in buffer (50 μl) [17] were typed using broad-spectrum polymerase chain reaction (PCR). HPV short PCR fragment 10 (SPF_10_) and PCR DNA enzyme immunoassay (PCR-DEIA) were used to amplify and hybridize with a cocktail of nine conservative probes to identify at least 57 HPV genotypes. DEIA positive- Line probe assay (LiPA) negative samples were denoted as ‘non-typeable/unidentifiable’ HPV types.

LiPA25 version 1 system (Labo Biomedical Products, Rijswijk, the Netherlands) was also used to genotype 25 HR and LR HPV types (14 HR [HPV-16, -18, -31, -33, -35, -39, -45, -51, -52, -56, -58, -59, -66 and -68] and 11 LR-HPV types [HPV-6, -11, -34, -40, -42, -43, -44, -53, -54, -70 and -74]) [18]. Sequence variation within the SPF_10_ inter-primer region did not allow a distinction between HPV types -68 and -73 [19].

All women completed two questionnaires which assessed health-related behavior and their awareness of HPV.

### Statistical analyses

The primary objective was to estimate the prevalence of any HPV-DNA and HPV types (including multiple infections) among Bahraini and non-Bahraini women aged ≥20 years attending clinics for routine cervical screening or those ≥16 years of age visiting clinics for post-natal check-up. The secondary objectives were to describe HPV type distribution by risk categories (including HR and LR types [20]) according to age and baseline characteristics and to understand health-related behaviors and HPV infection awareness in this population.

Based on an HPV prevalence rate of 11% in the Kingdom of Bahrain in 2006 [8], and allowing for 10% of non-evaluable women, based on cytological precision (2.5%–3.0%), the target enrolment was 460–660 women. Each age group (16–19, 20–24, 25–34, 35–44, 45–54 and ≥55 years) required a minimum of 75 women.

The percentage of HPV-positive women was tabulated with corresponding 95% confidence intervals (CI). Descriptive analyses on HPV prevalence, HPV-types, age distribution, potential risk factors (education level, age at first marriage, marital partners over life-time, parity and smoking status) and HPV status were performed.

An exploratory analysis was undertaken to study the association between the HPV status and nationality using adjusted odds ratio from a multiple logistic regression model and the association between risk factors and HPV prevalence using multivariate analysis. All statistical analyses were performed using the statistical analysis software (SAS®) version 9.2.

## Results

### Study population

Of 577 enrolled women, 571 were included in the final analysis (cervical samples from six were not collected or tested). The mean age (standard deviation) was 35.57 (±11.19) years and the majority (81.3%; 464/571) were Bahraini nationals (Table 1). From the data available from 553 women, 11 women were single, 513 were married 7 were divorced or separated and 20 women were widowed.

**Table 1:** **Baseline characteristics (N = 571)**

Characteristics	Parameters or Categories	Value or n	%
Age at diagnosis (Years)	N	571	-
	Mean	35.57	-
	SD	11.19	-
Nationality	Bahraini	464	81.3
	Others	107	18.7
Race	African heritage/African American	3	0.5
	Asia – Central/South East Asia	414	72.5
	White – Arabic/North African heritage	147	25.7
	White – Caucasian/European Heritage	5	0.9
	Other	2	0.4

N: Number of subjects enrolled; n: number of subjects in a given category; Value: value of the considered parameter; %: n / N × 100; SD: Standard deviation.

### HPV Overall prevalence and type distribution

HPV DNA was detected in 56 women (9.8%; 95% CI: 7.5–12.5). Among these, 28 (4.9%; 95% CI: 3.3–7.0) had single HPV-type infection and 15 (2.6%; 95% CI: 1.5–4.3) had multiple HPV-type infection. Thirteen women (2.3%; 95% CI: 1.2–3.9) were infected with unidentifiable HPV-types.

The most prevalent HR-HPV types were HPV-52 in eight women (1.4%) and HPV-16, -31 and -51 each in six women (1.1%). HR-HPV-18 was observed in two women (0.4%).

**Figure 1:**
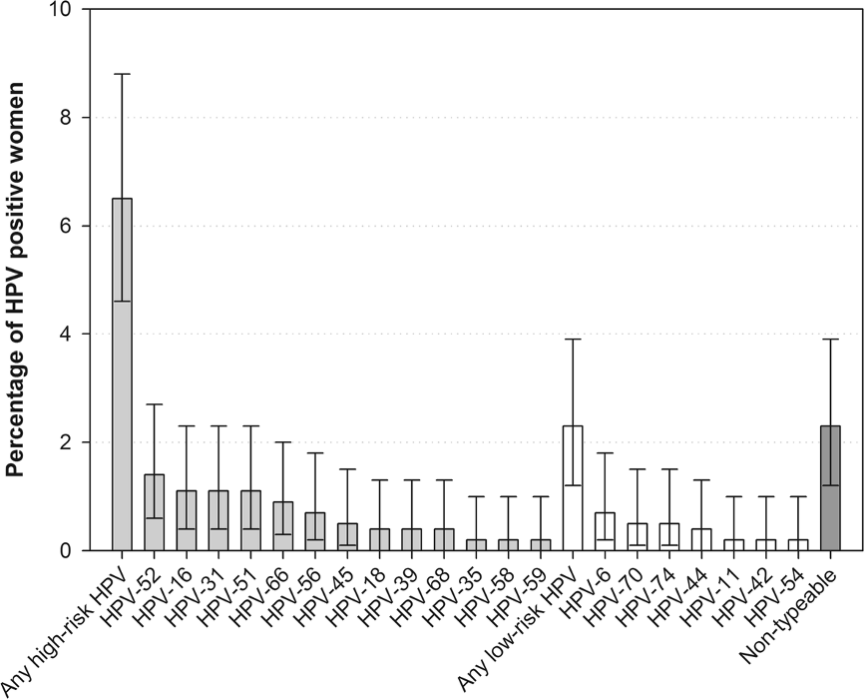
**Distribution of HPV types (N = 571).**

**Figure 2:**
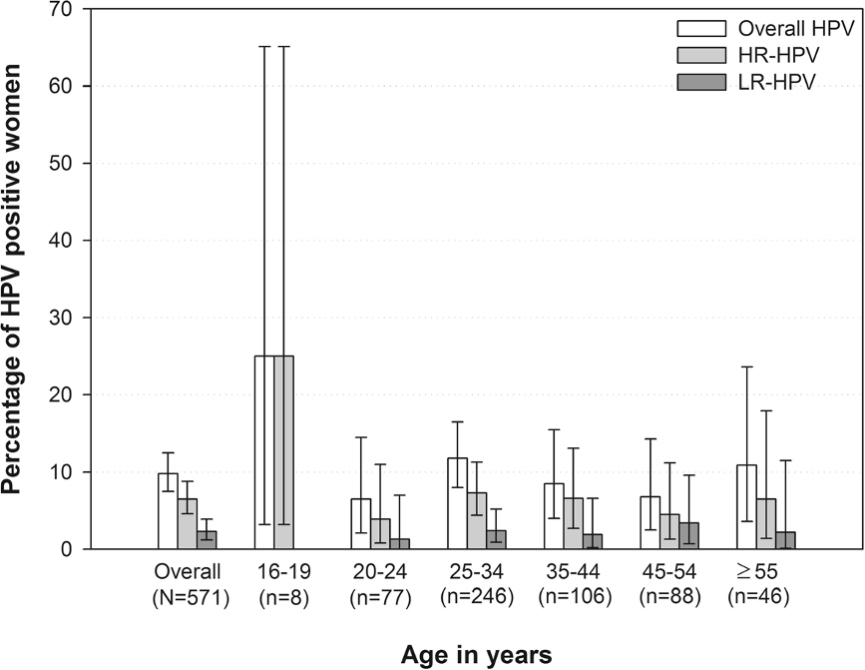
**Distribution of HR-HPV and LR-HPV types by age group (N = 571).**

The most prevalent LR types were HPV-6 in four women (0.7%) and HPV-70 and -74 each in three subjects (0.5%) (Figure 1). The prevalence of HPV was similar across all age groups (Table 2), HR-HPV was most frequently observed (25%) in women aged 16–19 years (Figure 2).

**Table 2:** **Distribution of HPV type infection by age group (N = 571)**

Age group	Overall (N=571)		16–19 (n’=8)		20–24 (n’=77)		25–34 (n’=246)		35–44 (n’=106)		45–54 (n’=88)		≥55 (n’=46)	
	n	95% CI (LL–UL)	n	95% CI (LL–UL)	n	95% CI (LL–UL)	n	95% CI (LL–UL)	n	95% CI (LL–UL)	n	95% CI (LL–UL)	n	95% CI (LL–UL)
HPV -	515	90.2 (87.5–92.5)	6	75 (34.9–96.8)	72	93.5 (85.5–97.9)	217	88.2 (83.5–92.0)	97	91.5 (84.5–96.0)	82	93.2 (85.7–97.5)	41	89.1 (76.4–96.4)
HPV +	56	9.8 (7.5–12.5)	2	25 (3.2–65.1)	5	6.5 (2.1–14.5)	29	11.8 (8.0–16.5)	9	8.5 (4.0–15.5)	6	6.8 (2.5–14.3)	5	10.9 (3.6–23.6)
Single infection	28	4.9 (3.3–7.0)	1	12.5 (0.3–52.7)	2	2.6 (0.3–9.1)	14	5.7 (3.1–9.4)	4	3.8 (1.0–9.4)	5	5.7 (1.9–12.8)	2	4.3 (0.5–14.8)
Multiple infection	15	2.6 (1.5–4.3)	1	12.5 (0.3–52.7)	1	1.3 (0–7.0)	7	2.8 (1.2–5.8)	3	2.8 (0.6–8.0)	1	1.1 (0–6.2)	2	4.3 (0.5–14.8)
Infection with unidentifiable HPV type*	13	2.3 (1.2–3.9)	0	0 (0–36.9)	2	2.6 (0.3–9.1)	8	3.3 (1.4–6.3)	2	1.9 (0.2–6.6)	0	0 (0–4.1)	1	2.2 (0.1–11.5)

HPV -: HPV Negative; HPV +: HPV Positive; N: total number of women included in the final analysis; n’: total number of women whose cervical samples were tested in each age strata; n: number of women in a given category; %: n / Number of women with available results × 100; 95% CI: exact 95% confidence interval; LL: Lower limit; UL: Upper limit.

### HPV DNA prevalence and type distribution among HPV positive women

Among the 56 HPV-positive women, infection with single, multiple and unidentifiable HPV type infection was observed in 50% (95% CI: 36.3–63.7; 28/56), 26.8% (95% CI: 15.8–40.3; 15/56) and 23.2% (95% CI: 13.0–36.4; 13/56), respectively.

Among those infected with HPV, 14.3% (8/56) women were infected with HR-HPV-52, followed by 10.7% (6/56) each with HR-HPV types -16, -31 and -51; HR-HPV-18 was detected in 3.6% women (2/56). The most prevalent LR-HPV types were LR-HPV-6 (7.1% [4/56]), and LR-HPV-70 and -74 (5.4% [3/56] each).

### HPV Co-infection

Three of the six women infected with HPV-16 (50%) were co-infected with other HR-HPV types (HPV-16/31, HPV-16/45 and HPV-16/56, respectively). Both HPV-18 infected women were co-infected with HR HPV-52.

### Risk factors and awareness

The percentage of Bahraini women positive for HPV as compared to the non-Bahraini women was 6.7% (31/464) vs. 23.4% (25/107), respectively. When other risk factors (age at sample collection, education level, number of marital partners, parity, smoking status) were adjusted, non-Bahraini women had a higher risk of HPV infection in comparison with Bahraini women (adjusted odds ratio: 3.7 [95% CI: 1.9–7.6]; p-value = 0.0002) (Table 3).

**Table 3:** **Prevalence of HPV by risk factors (N = 571)**

Risk factor	Categories	n	HPV+	%	Adj. OR	95% CI (LL–UL)	P-value
Age at sample collection (years)	<30*	207	21	10.1	-	-	
	30-39	187	19	10.2	0.94	0.41–2.14	0.8755
	40-49	91	9	9.9	1.35	0.49–3.68	0.5592
	50-60	75	6	8.0	1.09	0.33–3.60	0.8843
	>60	11	1	9.1	1.41	0.14–14.62	0.7731
Nationality	Non-Bahraini	107	25	23.4	3.7	1.9–7.6	0.0002
	Bahraini*	464	31	6.7	-	-	-
Education level	No formal education*	15	2	13.3	.	-	-
	Primary	59	5	8.5	0.51	0.08–3.50	0.4966
	Secondary	210	19	9.1	0.71	0.12–4.14	0.6992
	Post-secondary/University	266	30	11.3	0.69	0.12–3.93	0.6735
Number of marital partners	1*	511	46	9.0	-	-	-
	2-5	28	6	21.4	2.50	0.84–7.44	0.0986
Parity	0-2	263	30	11.4	.	-	-
	3-5	216	13	6.0	0.59	0.26–1.33	0.1998
	≥ 6	46	4	8.7	0.75	0.19–2.95	0.6780
Smoking status	No*	509	47	9.2	-	-	-
	Yes	43	9	20.9	1.18	0.41–3.40	0.7598

N: total number of women included in the final analysis; n: number of subjects in a given category; %: HPV+ / number of subjects with available results × 100; Adj. OR: Odds ratio adjusted for the other variables; 95% CI: Wald 95% confidence interval; LL: lower limit; UL: upper limit.

Note: *Reference category.

None of the studied risk factors were significantly associated with either HPV-16 or HPV-18 or any HR-HPV infection as identified from questionnaire data using multivariate logistic regression analysis.

HPV awareness questionnaire was collected to understand the level and accuracy of awareness regarding cause, transmission and prevention of HPV infection. Among the women who completed this questionnaire, 68.7% (369/537) had never heard about HPV. However 80.9% (432/534) of women believed that it is possible to prevent CC and the majority (91.3%, 495/542) showed an interest in vaccination (Table 4).

**Table 4:** **Summary of HPV infection awareness among women (N’ = 542)**

Characteristics	Categories	n	%
How frequent is cervical cancer in women?	Very frequent	40	7.4
	Frequent	162	29.9
	Rare	228	42.1
	Not sure	112	20.7
What do you think is/are the main reasons for cervical cancer?*	Abnormal cells growing inside the body	166	30.6
	Bacterial infection	62	11.4
	Viral infection	124	22.9
	None	45	8.3
	Not sure	153	28.2
Which among these can cause cervical cancer?*	Persistent infection with HPV	131	24.2
	Rous sarcoma virus	14	2.6
	Hereditary/genetic factors	160	29.5
	None	50	9.2
	Not sure	194	35.8
What do you think can turn into cervical cancer*	Genital warts	86	15.9
	Bacterial infection	63	11.6
	Viral infection	119	22.0
	Fungal infection	25	4.6
	None	57	10.5
	Not sure	195	36.0
Apart from avoiding unwanted pregnancy, what would you think can happen with using contraceptive pills*	Protects against cervical cancer	33	6.1
	Increases risk of cervical cancer	158	29.2
	No ill effect at all	159	29.3
	Not sure	192	35.4
Did you hear about HPV before?	Yes	168	31.3
	No	369	68.7
	Missing	5	-
If yes*,	General physician	76	14.0
	Friend or family member	27	5.0
	Internet	14	2.6
	TV/Magazine/Newspaper	30	5.5
	Other	3	0.6
How is HPV transmitted?*	Contaminated food/ Water	7	1.3
	Mosquito bite	2	0.4
	Sexually	344	63.5
	None	18	3.3
	Not sure	167	30.8
How is cervical cancer diagnosed?*	Pap smear test (Papanicolaou test)	139	25.6
	Colposcopy	24	4.4
	Biopsy sample testing (histological)	185	34.1
	All above	141	26.0
	None	3	0.6
	Not sure	53	9.8
Is it possible to prevent cervical cancer?	Yes	432	80.9
	No	34	6.4
	Not sure	68	12.7
	Missing	8	-
If yes*,	Through cancer vaccine	56	10.3
	Through responsible sexual behavior	116	21.4
	Through cervical screening	272	50.2
	Through condom use	13	2.4
If the vaccine against cervical cancer is available, would you be interested in getting vaccinated?	Yes	495	91.3
	No	47	8.7

N’: number of women for whom the questionnaire data was collected; n: number of women in specified category for whom the questionnaire data was collected; %: n/N*100.

*Participating women could have selected more than one option.

## Discussion

CC is associated with a considerable disease burden in the Kingdom of Bahrain and represents an important health concern among the female population [2–4]. This study provides a recent estimate of the prevalence and type distribution of both HR- and LR-HPV in Bahraini and non-Bahraini women from 16 years of age. The findings from our study suggest that nearly 10% of women in the Kingdom of Bahrain harbored HPV-DNA, which is consistent with previous estimates of 11% [8] and 12.1% [2]. This prevalence is higher than that reported in Kuwait (2.4%) [21] and Saudi Arabia (5.6%) [22], but is in within the 0–25% range reported for women with normal cytology across the extended Middle East and North Africa [23].

The most prevalent HPV types observed in our study: HR-HPV-52, -16, -31 and -51 and LR-HPV-6, -70 and -74 are consistent with worldwide estimates of circulating HPV types causing CC [7, 24]. Although previous reports indicate that HR-HPV-16 and -18 cause the majority of CC cases worldwide [7], in our study, the overall prevalence of HR-HPV-18 was very low (0.4%). However, since the number of women positive for HPV DNA itself was low (n = 56), our results need to be interpreted with caution.

The highest prevalence of HR-HPV types (25%) was observed in 16–19 year old women, which is in accordance with published worldwide meta-analyses [24, 25] which reported higher HR-HPV type prevalence among women younger than 25 years of age. Other studies from the extended Middle East and North Africa also support our findings, whereby HPV prevalence was highest after sexual debut (20–24 years) but decreased with age [23].

None of the risk factors assessed in our study (education level, age at first marriage, number of marital partners over life time, parity and smoking status) were associated with the presence of HR-HPV types including HPV-16 or HPV-18.

Among the women who completed the health-related behavior and awareness of HPV questionnaire, the majority (68.7%) had no prior knowledge of HPV. In a previous study of Egyptian women, only 1.5% of the urban population underwent routine cervical screening tests, indicating a low awareness level of HPV [26]. Although the vast majority (88.6%) of Bahraini population live in urban areas [2], it is clear that effective measures are needed to increase the awareness of HPV. The willingness of women to receive vaccination against HPV, as observed in this study, might support the measures to prevent HPV infection.

Bivalent and quadrivalent HPV vaccines have been shown to protect against HR-HPV-16 and -18 [10, 13]. Although both prophylactic HPV vaccines have been licensed in the Kingdom of Bahrain since 2009 [2], they have not been included in the national immunization program and their use is limited to private clinics. The estimated disease burden, prevalence and type distribution of HPV data from our study might therefore highlight the need to include prophylactic HPV vaccines which offer broader protection into routine immunization programs.

The main strengths of our study were: the inclusion of both Bahraini and non-Bahraini citizens, enabling an assessment of HPV prevalence among the entire population; and the absence of age group restriction, which allowed us to study a wide age-range. Furthermore, the primary healthcare centers and the hospital were recognized by the Ministry of Health as reference hospitals. The population visiting these centers represented 90% of the local population and our results are representative of the Kingdom of Bahrain considering we met estimated sample size to determine the HPV prevalence in the target population (including nationals and non-national women). The possibility of selection bias is acknowledged in our study as there are some differences between Bahraini and non-Bahraini women to be considered. Most of non-Bahraini are married expatriate, while single females stay only for few years and were more unlikely to be enrolled in the study. However, all women were invited to participate regardless of their nationality and there were no differences in the acceptance rate between nationals and non-nationals identified. Furthermore, the primary healthcare centers were recognized by the Ministry of Health as reference hospitals and there are no differences reported in the use and access for health services, especially for post-natal and screening among women in the country. Lastly, considering the design of the study these results correspond to a single point of time and as HPV infections may be transient and spontaneously resolve [27], the prevalence of HPV might therefore vary with time.

## Conclusion

The overall prevalence of HPV in Bahrain was 9.8%. The most common HR-HPV types were -52, -16, -31 and -51 and LR-HPV types -6, -70 and -74. The data presented in our study might help healthcare authorities determine the impact of introducing preventive measures, such as prophylactic vaccination, to reduce the burden of CC in the Kingdom of Bahrain.

### Trademark

*Cervarix* is a trademark of the GlaxoSmithKline group of companies.

*Gardasil* is a trademark of Merck & Co. Inc.

*Thinprep* is a trademark of Hologic, Inc.

## Abbreviations

CC: Cervical Cancer
CI: Confidence Interval
DEIA: DNA Enzyme Immunoassay
HPV: Human Papillomavirus
HR: High-Risk
LiPA25: Line Probe Assay 25
LR: Low-Risk
PCR: Polymerase Chain Reaction
SPF-10: Short PCR Fragment 10.
